# Protein-driven inference of miRNA–disease associations

**DOI:** 10.1093/bioinformatics/btt677

**Published:** 2013-11-21

**Authors:** Søren Mørk, Sune Pletscher-Frankild, Albert Palleja Caro, Jan Gorodkin, Lars Juhl Jensen

**Affiliations:** ^1^Center for non-coding RNA in Technology and Health, ^2^Department of Veterinary Clinical and Animal Sciences, ^3^Department of Disease Systems Biology, Novo Nordisk Foundation Center for Protein Research and ^4^The Novo Nordisk Foundation Center for Basic Metabolic Research, University of Copenhagen, Denmark

## Abstract

**Motivation:** MicroRNAs (miRNAs) are a highly abundant class of non-coding RNA genes involved in cellular regulation and thus also diseases. Despite miRNAs being important disease factors, miRNA–disease associations remain low in number and of variable reliability. Furthermore, existing databases and prediction methods do not explicitly facilitate forming hypotheses about the possible molecular causes of the association, thereby making the path to experimental follow-up longer.

**Results:** Here we present miRPD in which miRNA–Protein–Disease associations are explicitly inferred. Besides linking miRNAs to diseases, it directly suggests the underlying proteins involved, which can be used to form hypotheses that can be experimentally tested. The inference of miRNAs and diseases is made by coupling known and predicted miRNA–protein associations with protein–disease associations text mined from the literature. We present scoring schemes that allow us to rank miRNA–disease associations inferred from both curated and predicted miRNA targets by reliability and thereby to create high- and medium-confidence sets of associations. Analyzing these, we find statistically significant enrichment for proteins involved in pathways related to cancer and type I diabetes mellitus, suggesting either a literature bias or a genuine biological trend. We show by example how the associations can be used to extract proteins for disease hypothesis.

**Availability and implementation:** All datasets, software and a searchable Web site are available at http://mirpd.jensenlab.org.

**Contact:** lars.juhl.jensen@cpr.ku.dk or gorodkin@rth.dk

## 1 INTRODUCTION

Since the initial discovery of microRNAs (miRNAs) 20 years ago, (Lee *et al.*, 1993; Wightman *et al.*, 1993), the number of known miRNAs has grown to thousands of currently annotated miRNAs from a wide variety of species [e.g. the inventory of miRBase (Kozomara and Griffiths-Jones, 2011)]. MicroRNAs are increasingly being recognized as key regulatory players (Friedman *et al.*, 2009; Lim *et al.*, 2005), and dysregulation of them is hence an obvious source of aberrant cell behavior. Not surprisingly, miRNAs have been associated with a large number of diseases (Esteller, 2011; Mendell and Olson, 2012) including schizophrenia (Feng *et al.*, 2009), cardiovascular diseases (Small and Olson, 2011) and cancer (He *et al.*, 2005; Võsa *et al.*, 2011).

MicroRNAs function by base pairing with 3′-UTRs of messenger RNAs (mRNAs), triggering their translational repression or degradation (Ambros, 2004; Bartel, 2004, 2009; Meister and Tuschl, 2004). The targeting depends on either complete sequence complementarity for inducing transcript degradation or partial sequence complementarity for translational repression. In addition to the base pairing between the miRNA and the mRNA, targeting also depends on the local sequence context of the target site and on a number of proteins participants (Grimson *et al.*, 2007). Owing to this complexity, miRNA target prediction remains a significant computational challenge, although advances in recent years have considerably improved the reliability with which miRNA::mRNA interactions (from now on referred to as miRNA–protein associations) can be predicted (Betel *et al.*, 2010; Garcia *et al.*, 2011; Kertesz *et al.*, 2007; Krek *et al.*, 2005). Moreover, experimental validation of miRNA target predictions is difficult and the number of functionally verified targets sites remains low (Kuhn *et al.*, 2008).

Because of their potential for wide-spread involvement in diseases, a number of resources have emerged containing experimentally verified miRNA–disease associations obtained via text mining (Dweep *et al.*, 2011; Jiang *et al.*, 2009; Lu *et al.*, 2008; Ruepp *et al.*, 2010; Yang *et al.*, 2010). However, the majority of miRNA–disease associations has presumably not been discovered yet and thus cannot be mined from the literature. In concordance with this, *de novo* prediction of miRNA–disease associations is receiving increasing attention (Chen and Zhang, 2013; Chen *et al.*, 2012; Jiang *et al.*, 2010; Rossi *et al.*, 2011; Xiao *et al.*, 2012; Xu *et al.*, 2013).

To our knowledge, none of the existing methods for predicting miRNA–disease associations provide any biological hypothesis underpinning the predictions. Therefore, we here present an approach in which miRNAs are linked to diseases via proteins, thereby directly providing biological hypotheses. Specifically, we infer miRNA–disease associations by network analysis of known or predicted miRNA–protein associations and text-mined protein–disease associations. To account for the variable reliability of both types of associations, we provide a scoring scheme that allows for ranking of the inferences by confidence.

## 2 MATERIALS AND METHODS

### 2.1 miRNA–protein associations

We use miRNA–protein associations from three sources: a set of text mining-based miRNA-target associations from Croft *et al.* (2012) and predictions from two state-of-the-art miRNA-target methods, namely, MiRanda version 5 (Betel *et al.*, 2010) and TargetScan version 6.2 (Garcia *et al.*, 2011). To enable comparison and integration of these sources, we mapped all miRNAs to miRbase identifiers and all targets to Ensembl protein identifiers (ENSPs) using the STRING aliases file (Franceschini *et al.*, 2013). Each association between a miRNA (*M*) and a protein (*P*) has a quality measure assigned to it, referred to as 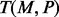. In case of the manually curated Croft dataset, we used as score the number of Medline abstracts supporting the association. For MiRanda and TargetScan, we used the mirSVR and Context+ scores, respectively. In the small proportion of cases where MiRanda or TargetScan predicts multiple target sites for the same miRNA within a single mRNA, we sum their scores. Table 1 summarizes the miRNA–protein association datasets.

**Table 1.** Sources of miRNA–protein associations

Dataset	Data type	Pairs	miRNAs	Proteins	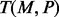
Croft	Curated	146	49	127	Number of abstracts
MiRanda	Predictions	630 373	711	16 518	mirSVR
TargetScan	Predictions	502 064	1537	14 190	Context+

### 2.2 Protein–disease associations

To obtain protein–disease associations to be used for prediction of miRNA–disease associations, we downloaded the complete dataset from the DISEASES database (http://diseases.jensenlab.org; S.P.F., A.P.C. og L.J.J., manuscript in preparation). To identify proteins and diseases mentioned in Medline abstracts, this resource makes use of the efficient tagger described in Pafilis *et al.* (2013) to identify names of human proteins from the STRING database (Franceschini *et al.*, 2013) and disease names from the Disease Ontology (Schriml *et al.*, 2012). The protein–disease associations were automatically mined from Medline abstracts and have quality scores assigned to them. The scores are computed using a slightly modified version of the co-occurrence-based text-mining scores in STRING v9.1 (Franceschini *et al.*, 2013), which for completeness is outlined as follows.

The scoring scheme takes into account co-occurrences within an abstract and within individual sentences of the abstract and combines them in a weighting scheme. First, a weighted count (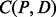) is calculated for each pair of a protein *P* and a disease *D* over *n* abstracts:

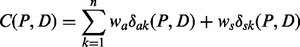

where 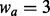 and 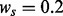 are the weights for co-occurrence within the same abstract and the same sentence, respectively. The delta functions 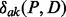 and 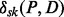 are 1, if *P* and *D* are co-mentioned in abstract *k* or a sentence therein. Based on the weighted counts, the co-occurrence score [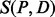] is defined as follows:

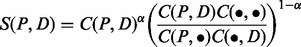

where 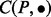 is the sum over diseases paired with protein *P*, 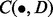 is the sum over all proteins paired with disease *D* and the normalizing factor 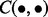 is the sum over all pairs of proteins and diseases. The weighting factor 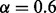.

Because these scores will change both with the growth of Medline and disease terms in the Disease Ontology, we convert them into the more robust and easier to interpret *Z*-scores (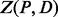) relative to a background distribution. To this end, we assume that the empirically observed score distribution is a mixture of lower-scoring random background and the higher-scoring true signal. We model the background distribution as a Gaussian and estimate its mean as the mode of the mixture distribution. Because we have empirically observed that the 40th percentile in this case coincides with the mode, we estimate the variance based on the distance between the 20th and 40th percentiles.

Finally, we filtered out associations involving 1992 broad disease concepts from the Disease Ontology to obtain a set of 234 834 scored protein–disease associations among 14 871 proteins and 2586 diseases.

In addition to the protein–disease associations derived from text mining, we have used two other sources of protein–disease associations. One is derived from Uniprot, the other from The Genetics Home Reference (GHR). Both datasets have been mapped to Disease Ontology terms and filtered for the same generic disease terms as the text mining-derived protein–disease associations. The protein–disease associations from Uniprot and GHR are unscored and are hence given a score of 1 such as to be used with our scoring schemes. The Uniprot-based dataset consists of 1632 proteins and 161 diseases in 3469 associations. The GHR dataset consists of 950 proteins and 468 diseases in 2509 associations.

### 2.3 Inference of miRNA–disease associations

To infer miRNA–disease associations and rank them by confidence, we need a scoring scheme that combines the miRNA–protein association scores, 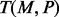, and protein–disease association scores, 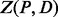.

Let 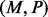 denote the association between miRNA *M* and protein *P*, and let *P_M_* denote the set of proteins associated with *M*. Correspondingly, let 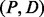 denote the association between protein *P* and disease *D*, and let *P_D_* denote the set of all proteins associated to disease *D*. We can then define a score between miRNA *M* and disease *D* as follows:

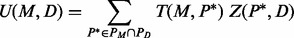

where 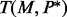 and 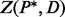 are the already described miRNA–protein and protein–disease association scores, respectively.

As an alternative scoring function, we used only the best scoring protein connection between a miRNA and a disease instead of the sum over all connections:

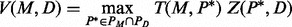



For each of the two scoring functions and for each of the three sets of miRNA–protein associations (Table 1), we produced a list of inferred miRNA–disease associations ranked by score. The scoring schemes use the product of the two subscores in order not to affect the rank of the final scored miRNA–disease associations due to differences in scale among the subscores. These six ranked lists can be downloaded from http://mirpd.jensenlab.org.

### 2.4 Prediction performance evaluation

To benchmark our method for predicting miRNA–disease associations, we used the manually curated gold standard set of direct associations between miRNAs and diseases from Jiang *et al.* (2010). To allow for direct comparison with the datasets described earlier in the text, we mapped the miRNAs and diseases to miRbase and Disease Ontology identifiers, respectively. After mapping, the set consisted of 236 associations among 92 miRNAs and 48 diseases.

For each of the six ranked lists of inferred miRNA–disease associations, we first disregarded all associations that involved an miRNA or a disease not at all present in the gold standard. Next we ranked the remaining inferred associations in decreasing order by their scores, 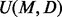 and 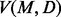, respectively. To measure the agreement with the gold standard, we calculated the cumulative number of gold standard associations identified as function of rank.

**Fig. 1.**
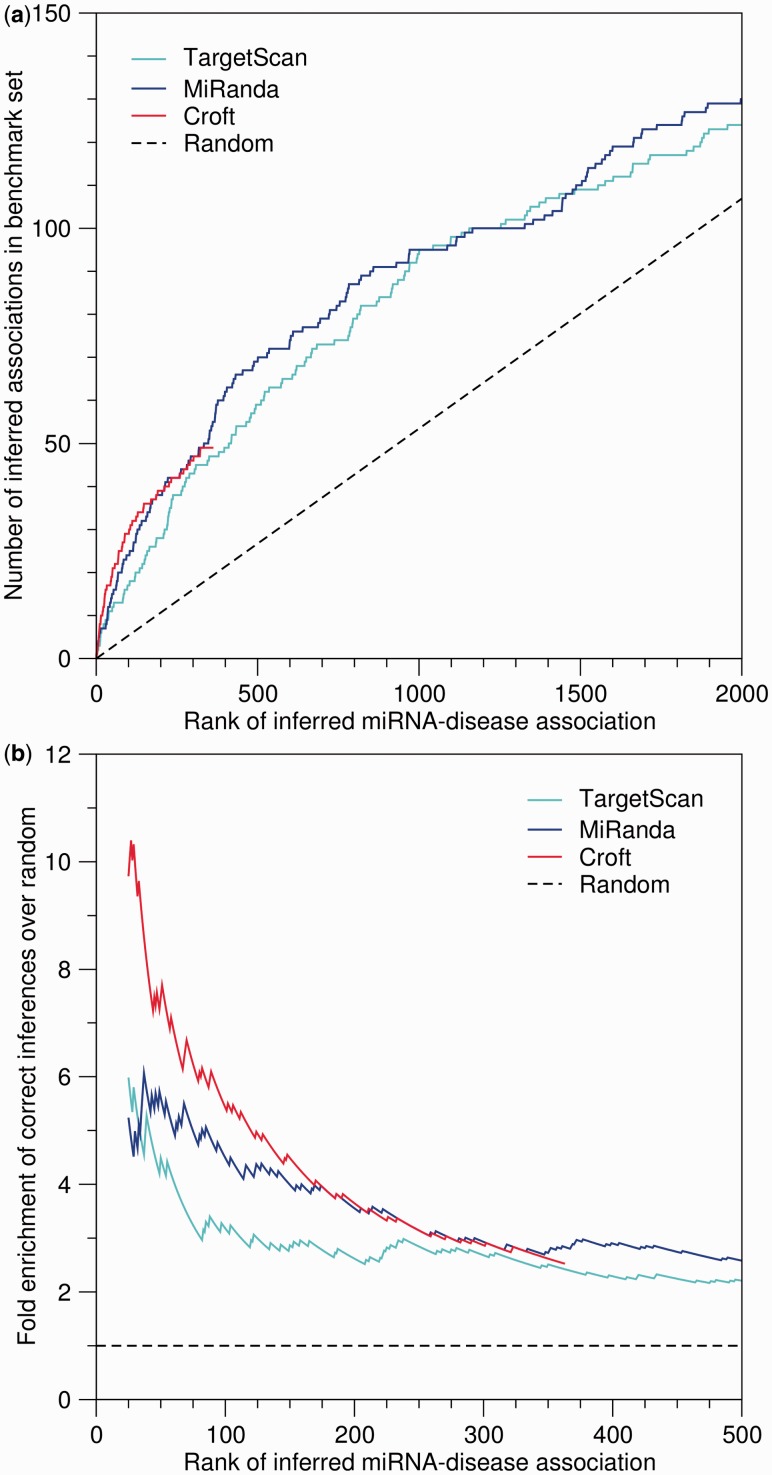
Benchmarking the quality of inferred miRNA–disease associations. The miRNA–disease associations inferred from three sets of miRNA–protein associations were ranked according to the scores 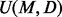. (**a**) Number of correct miRNA–disease associations obtained according to the gold standard from Chen and Zhang (2013) as a function of rank. (**b**) Fold enrichment of correct miRNA–disease associations over the expectation from a random background model. We only show enrichments starting from rank 25, as the counts are too low to reliably estimate the enrichment below this rank. Notice that only predicted miRNA–disease associations where the miRNA or the disease is present in the benchmark dataset is presented here, resulting in fewer data points than in the full prediction sets

To show that the method is better than random selection of miRNA–disease pairs, we as background use equiprobable sampling of all possible pairs that can be made from the miRNAs and diseases in the gold standard. In Figure 1 this corresponds to a straight line with a slope of 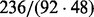.

### 2.5 Pathway enrichment analysis

To analyze if the proteins that mediate miRNA–disease associations are predominantly involved in certain biological pathways, we started from the three sets of medium-confidence miRNA–protein–disease associations described in Section 3. Because some of the multiple proteins that together support a certain miRNA–disease association may contribute little, we focused the analysis on the set of proteins that gives the highest single contribution to at least one miRNA–disease association. This resulted in 93 922 and 376 unique proteins for the Croft-, MiRanda- and TargeScan-based datasets, respectively. For use in the statistical analysis, we further compiled a list of the 14 871 proteins involved in protein–disease associations according to the text mining of Medline abstracts described earlier in the text. On these lists we used the DAVID tool (Huang *et al.*, 2009a, b) to identify statistically significantly enriched KEGG pathways (Kanehisa *et al.*, 2012) for each of the three lists of proteins connecting miRNAs and diseases relative to the background set. We also used DAVID to identify enriched Gene Ontology terms, which gave results consistent with the pathway analysis (data not shown). Owing to the sparsity of the protein–disease associations derived from Uniprot and GHR, only few (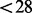) of the benchmark miRNA–disease associations are of miRNAs or diseases that are present in the input data, hindering reliable prediction performance evaluation.

## 3 RESULTS

### 3.1 Resource of miRNA–protein–disease associations

We have combined three sets of curated and predicted miRNA–protein association approaches with text-mined protein–disease associations and scored the resulting miRNA–disease associations using the two scoring schemes described in Section 2 [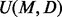 and 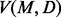]. To make the data easily available for use by other researchers, we have set up a web resource (http://mirpd.jensenlab.org) that enables users to search, e.g. for a certain miRNA.

### 3.2 Assessment of inferred associations

To assess the quality of the associations in the resource, we compare the miRNAs–protein–disease associations to the small gold standard set of miRNA–disease associations from Chen and Zhang (2013). Because the latter set presumably is only a tiny fraction of the actual miRNA–disease associations, it is impossible to estimate the number of false positives and hence the positive predictive value. However, because it is possible to estimate the number of true positives, we can plot this as function of the total number of positive predictions and compare it with the expectation from a random background model (Fig. 1a).

Figure 1a shows that irrespective of the source of miRNA–protein associations, the inferred miRNA–protein–disease associations capture more of the miRNA–disease associations from the gold standard than expected at random. Unsurprisingly, the reliability of the inferred associations depends on the quality of the miRNA–protein associations. The most reliable inferred associations are obtained when using the manually curated set of miRNA–protein associations (Croft *et al.*, 2012); however, this also results in considerably fewer associations than when making use of predicted miRNA–protein associations. We also see that MiRanda predictions result in a slightly better ranking than that of TargetScan predictions. Using the alternative scoring scheme 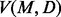 gave comparable but worse results (data not shown).

An alternative way to plot the data is to instead plot the fold enrichment of correct miRNA–disease associations over the expectation from a random background model (Fig. 1b). The major advantage of using fold enrichment is that it can be accurately estimated using even an incomplete gold standard set.

Consistent with Figure 1a, the highest fold enrichment of up to 10 is seen for the top-ranked inferences based on the manually curated miRNA–protein associations from Croft *et al.* (2012). Inferences based on MiRanda and TargetScan both give up to 6-fold enrichment, with the fold enrichment for the MiRanda-based inferences dropping off slower than for the TargetScan-based ones.

For convenience, we define high-confidence and medium-confidence subsets of the miRNA–protein–disease associations inferred from the Croft-, MiRanda- and TargetScan-based miRNA–protein associations. From inspection of Figure 1b, we decided to use the score cutoffs corresponding to 5-fold and 3.5-fold enrichment to define the high-confidence and medium-confidence sets, respectively. In total the medium-confidence sets contain 14 599 miRNA–protein–disease associations among 1169 miRNAs, 1570 proteins and 738 disease terms. These filtered as well as the complete unfiltered lists of inferred miRNA–protein–disease associations are available for download at http://mirpd.jensenlab.org.

### 3.3 Functional categorization of intermediate proteins

To characterize the functions, proteins that mediate the inference of miRNA–disease associations, we focused on the proteins within the three sets of medium-confidence associations that most strongly connect the miRNAs and diseases (see Section 2 for details). For each of these, we identified statistically significantly pathways from the KEGG database (Kanehisa *et al.*, 2012), which are listed in Table 2. The lists are dominated by pathways for various forms of cancer as well as signal-transduction pathways known to play important roles in cancer such as ErbB and p53 signaling.

**Table 2.** Statistically enriched KEGG pathways among proteins connecting miRNAs to diseases

KEGG pathway	Number of proteins	*P*-value
Croft (93 proteins)		
Bladder cancer	9	1.7E-6
Pathways in cancer	18	3.8E-6
ErbB signaling pathway	9	1.8E-4
Prostate cancer	9	1.7E-4
Pancreatic cancer	8	3.2E-4
Chronic myeloid leukemia	8	3.2E-4
Melanoma	7	2.1E-3
Endometrial cancer	6	3.7E-3
Non-small cell lung cancer	6	3.9E-3
Small cell lung cancer	7	3.7E-3
Glioma	6	6.1E-3
MiRanda (922 proteins)		
Pathways in cancer	89	5.5E-14
Bladder cancer	25	5.8E-11
Hematopoietic cell lineage	32	3.8E-8
Cytokine–cytokine receptor interaction	63	3.1E-8
Chronic myeloid leukemia	27	1.8E-6
Colorectal cancer	29	1.7E-6
Prostate cancer	30	1.5E-6
Asthma	14	7.5E-6
Pancreatic cancer	25	1.0E-5
Glioma	22	3.6E-5
Endometrial cancer	19	1.2E-4
Complement and coagulation cascades	22	1.2E-4
Viral myocarditis	21	1.5E-4
Non-small cell lung cancer	19	1.8E-4
Melanoma	22	2.6E-4
Autoimmune thyroid disease	16	3.2E-4
p53 signaling pathway	21	3.1E-4
Hypertrophic cardiomyopathy	23	4.9E-4
ErbB signaling pathway	24	5.1E-4
Type I diabetes mellitus	14	5.4E-4
Jak-STAT signaling pathway	34	6.1E-4
Acute myeloid leukemia	18	7.5E-4
Renal cell carcinoma	20	1.5E-3
Primary immunodeficiency	13	1.5E-3
Intestinal immune network for IgA production	15	1.6E-3
Allograft rejection	12	1.7E-3
Focal adhesion	40	1.7E-3
Small cell lung cancer	22	2.0E-3
Dilated cardiomyopathy	22	3.2E-3
Thyroid cancer	11	5.0E-3
B cell receptor signaling pathway	19	5.7E-3
Maturity onset diabetes of the young	10	6.0E-3
T cell receptor signaling pathway	24	5.8E-3
TargetScan (376 proteins)		
Pathways in cancer	86	8.1E-39
Colorectal cancer	30	1.6E-15
Bladder cancer	22	5.0E-15
Prostate cancer	30	3.7E-15
Melanoma	27	6.0E-15
Glioma	25	2.0E-14
Pancreatic cancer	26	7.9E-14
Chronic myeloid leukemia	25	1.5E-12
Focal adhesion	38	3.0E-11
Renal cell carcinoma	21	2.1E-9
ErbB signaling pathway	22	1.6E-8
Endometrial cancer	17	3.8E-8
Non-small cell lung cancer	17	6.6E-8
Acute myeloid leukemia	16	8.3E-7
Thyroid cancer	12	8.2E-7
p53 signaling pathway	17	1.5E-6
Small cell lung cancer	18	7.6E-6
Cytokine–cytokine receptor interaction	33	9.5E-6
MAPK signaling pathway	32	5.6E-5
Neurotrophin signaling pathway	20	8.0E-5
Adherens junction	15	1.3E-4
Cell cycle	19	2.6E-4
T cell receptor signaling pathway	17	4.5E-4
Gap junction	14	2.4E-3
mTOR signaling pathway	10	5.0E-3
Hematopoietic cell lineage	13	6.2E-3
VEGF signaling pathway	12	6.1E-3
Apoptosis	13	6.4E-3
Viral myocarditis	11	6.3E-3
Melanogenesis	14	6.8E-3
Type I diabetes mellitus	8	7.9E-3
Regulation of actin cytoskeleton	22	8.9E-3

The DAVID tool (Huang *et al.*, 2009a, b) was used to identify statistically significantly enriched KEGG pathways (Kanehisa *et al.*, 2012) for each of the three medium-confidence sets of miRNA–protein–disease associations. The *P*-values listed have been corrected for multiple testing using the Benjamini–Hochberg procedure, and all pathways with a corrected *P*-value of 1E-3 or better are shown.

Besides cancer-related pathways, the analyses of both the MiRanda- and TargetScan-based miRNA–protein–disease associations show an enrichment for proteins involved in *Type I diabetes mellitus* as well as *viral myocarditis*. Existing literature already suggests a role for miRNAs in both of these diseases (Poy *et al.*, 2004; Xu *et al.*, 2012); however, our method expands on this by directly suggesting which proteins may mediate a certain miRNA–disease association.

### 3.4 Case study: miRNA-181 and diabetes mellitus

The pathway analysis revealed a statistically significant enrichment for proteins involved in the type I diabetes mellitus pathway. To demonstrate the ability of our miRNA–protein–disease associations to pinpoint potentially causal proteins, we examined the links between miRNAs and diabetes in more detail.

For the medium-confidence miRNA–disease associations based on the TargetScan miRNA–protein associations, 64 miRNAs are associated with diabetes mellitus. Of these, the four members of the miR-181 family (miR-181a, miR-181b, miR-181c and miR-181d) stand out, ranking fifth to eight most diabetes-related miRNAs in that prediction set. MicroRNA-181 is known from the literature to be associated with diabetes (Li *et al.*, 2011).

The protein that most strongly connects miR-181 to diabetes mellitus is glutamate decarboxylase 2, which is one of the eight proteins also found on the type I diabetes mellitus pathway (Table 2). Another protein that stands out is sirtuin-1, which ranks 9th, 9th, 7th and 8th for miR-181a, miR-181b, miR-181c and miR-181d, respectively. It is a nicotinamide adenine dinucleotide-dependent deacetylase that acts as a positive regulator of insulin signaling (Liang *et al.*, 2009). Moreover, it has been shown that downregulation of miR-181a upregulates sirtuin-1 and increases insulin sensitivity in hepatic cells (Zhou *et al.*, 2012).

This illustrates that using the protein-driven miRNA–disease associations not only reveals potentially new miRNAs involved in diseases but also provides candidate proteins as molecular hypotheses underpinning the associations, which can be tested, e.g. through knockdown of the mRNA or mutagenesis of the miRNA target region.

## 4 DISCUSSION

Whereas the awareness of miRNA–disease associations is growing, existing methods for identifying such associations falls in two broad categories: (i) text mining and curation of direct associations from literature and (ii) machine learning-based prediction methods. Generally, these approaches do not consider or attempt to identify the proteins that presumably mediate most of the interactions between miRNAs and diseases. Consequently, these studies contain relative few pointers for how further experimental analysis of specific cases (predictions) can be carried out. We meet this challenge by developing a method, miRPD, which explicitly includes the protein link between miRNA and disease. Even though this protein-driven approach overall result in fewer miRNAs and diseases than previously published studies, having the protein as an explicit part of the output readily allows the researcher to take up more far more explicit actions toward design of experiments.

We have showed that our approach, which explicitly combines miRNA–protein and protein–disease associations, results in miRNA–disease associations of much better quality than that of a random background. This was observed consistently for three sets of miRNA–protein associations: a hand-curated set of miRNA targets and the two popular target predictions methods MiRanda and TargetScan. In all three cases, we were able to obtain a *high-confidence set* of miRNA–protein–disease associations for which the rankings held a 5-fold enrichment over random. We similarly defined three *medium-confidence sets* with 3.5-fold enrichment.

Our resource (http://mirpd.jensenlab.org) provides a search interface for the three medium-confidence sets of 14 599 miRNA–protein–disease associations, connecting 1169 miRNAs to 738 diseases through 1570 proteins, that is automatically updated whenever new protein–disease associations become available, and features hyperlink for the protein entries to http//:diseases.jensenlab.org. This was exemplified by a case study of the involvement of miR-181 and diabetes mellitus, which revealed glutamate decarboxylase 2 and sirtuin-1 as likely causal molecular links between the miRNA and the disease.

Analyzing the proteins that most strongly connect miRNAs to diseases, we found a strong enrichment for cancer-related KEGG pathways. This can be due to (i) study bias in the miRNA field, or (ii) miRNAs actually being more involved in cancer than in other diseases. The latter is consistent with cancer being a disease of cellular regulation.

To our knowledge, we here provide the first resource of miRNA–disease associations, which explicitly lists proteins that are likely to mediate the association. The perspectives for further development of the method include taking into account expression data, combining target prediction methods into a single scoring scheme and including, e.g. other types of data such as co-expression and CliP-Seq.
